# AMPK alleviates high uric acid-induced Na^+^-K^+^-ATPase signaling impairment and cell injury in renal tubules

**DOI:** 10.1038/s12276-019-0254-y

**Published:** 2019-05-22

**Authors:** Jing Xiao, Sibo Zhu, Haochen Guan, Yuqi Zheng, Fengqin Li, Xiaoli Zhang, Hui Guo, Xiaojun Wang, Zhibin Ye

**Affiliations:** 1 Department of Nephrology, Huadong Hospital Affiliated to Fudan University, Shanghai, People’s Republic of China; 2 Shanghai Key Laboratory of Clinical Geriatric Medicine, Huadong Hospital Affiliated to Fudan University, Shanghai, People’s Republic of China; 3 School of Life Sciences, Fudan University, Shanghai, People’s Republic of China

**Keywords:** Acid, base, fluid, electrolyte disorders, Molecular biology

## Abstract

One of the mechanisms in hyperuricemia (HUA)-induced renal tubular injury is the impairment of Na^+^-K^+^-ATPase (NKA) signaling, which further triggers inflammation, autophagy, and mitochondrial dysfunction and leads to cell injury. Here, we used RNA sequencing to screen the most likely regulators of NKA signaling and found that the liver kinase B1(LKB1)/adenosine monophosphate (AMP)-activated protein kinase (AMPK)/ mammalian target of rapamycin (mTOR) pathway was the most abundantly enriched pathway in HUA. AMPK is a key regulator of cell energy metabolism; hence, we examined the effect of AMPK on HUA-induced dysregulation of NKA signaling and cell injury. We first detected AMPK activation in high uric acid (UA)-stimulated proximal tubular epithelial cells (PTECs). We further found that sustained treatment with the AMPK activator 5-aminoimidazole-4-carboxamide 1-β-d-ribofuranoside (AICAR), but not the AMPK inhibitor Compound C, significantly alleviated UA-induced reductions in NKA activity and NKA α1 subunit expression on the cell membrane by reducing NKA degradation in lysosomes; sustained AICAR treatment also significantly alleviated activation of the NKA downstream molecules Src and interleukin-1β (IL-1β) in PTECs. AICAR further alleviated high UA-induced apoptosis, autophagy, and mitochondrial dysfunction. Although AMPK activation by metformin did not reduce serum UA levels in hyperuricemic rats, it significantly alleviated HUA-induced renal tubular injury and NKA signaling impairment in vivo with effects similar to those of febuxostat. Our study suggests that AMPK activation may temporarily compensate for HUA-induced renal injury. Sustained AMPK activation could reduce lysosomal NKA degradation and maintain NKA function, thus alleviating NKA downstream inflammation and protecting tubular cells from high UA-induced renal tubular injury.

## Kidney damage: Identifying mechanisms behind uric acid effects

High serum levels of uric acid cause kidney tissue damage through cellular processes that have now been identified by researchers in China. Uric acid is a common component of urine, but causes damage if it is present in high levels in the blood (hyperuricemia). While investigating the mechanisms behind hyperuricemia, Zhibin Ye and co-workers at Fudan University in Shanghai recently showed that impairment of the Na^+^-K^+^-ATPase (NKA) signaling pathway, which regulates uric acid transportation through the kidneys, is a crucial feature of renal damage progression. The team have now shown that NKA is regulated by the AMP-activated protein kinase (AMPK) pathway, and that AMPK is enriched during the initial phases of hyperuricemia. Studies on rat models indicated that sustained AMPK activation restored NKA signaling, limiting damage from hyperuricemia.

## Introduction

Hyperuricemia (HUA) has recently been recognized as an independent risk factor for renal injury^1,2^. Renal tubulointerstitial injuries are frequently detected in urate nephropathy^3,4^. The renal proximal tubule is responsible for almost all renal urate transport and is the primary location for urate reabsorption^5^. We previously found that impaired activity and subcellular expression of Na^+^-K^+^-ATPase (NKA), the driver of urate transport on the basolateral side of proximal tubular epithelial cells (PTECs), lead to increased cellular autophagy; apoptosis; mitochondrial dysfunction; reduced intracellular ATP levels; sequential NKA-dependent activation of Src, Nod-like receptor pyrin domain-containing protein 3 (NLRP3), and interleukin-1β (IL-1β); and deregulation of urate transporters in HUA-induced renal tubular injury. These effects suggest that cellular metabolic disorder of such an ATPase could transfer stress signals to cause downstream inflammatory injury, leading to the deregulation of urate transportation and ultimately enhancing the renal injury of hyperuricemia^6^. However, whether alleviation of cell metabolic disorder can attenuate NKA-dependent HUA-induced renal tubular injury is currently unknown.

To screen possible target pathways involved in HUA-induced renal tubular injury, we performed RNA-seq on high uric acid (UA)-treated and untreated PTECs and found that adenosine monophosphate (AMP)-activated protein kinase (AMPK) was highly elevated upon UA treatment. AMPK is the most sensitive cell energy metabolism regulator^7^. AMPK activation via phosphorylation at Thr172 reduces energy consumption and cell activity and increases energy production (e.g., by increasing mitochondrial biogenesis to generate ATP)^8^, thus regulating the synthesis and decomposition of metabolic pathways to guarantee the cellular energy supply^9^. The roles of multiple signaling pathways downstream of AMPK in the pathogenesis of renal disease have been explored^9^. Moreover, AMPK regulates multiple ion channels in renal tubular epithelial cells^10^. In fact, an AMPK activator has been shown to preserve basolateral localization of NKA in Madin-Darby canine kidney cells subjected to energy depletion^11^ and to activate NKA activity in skeletal muscle cells^12^; however, the effect of AMPK on NKA signaling in HUA-induced renal tubular injury is currently unknown. Therefore, we hypothesized that AMPK might alleviate HUA-induced renal tubular NKA dysfunction and signaling impairment and thus subsequently reduce HUA-induced NKA-dependent inflammation and renal tubular cell injury. We first studied the effect of UA on AMPK activation in cultured PTECs. We then examined the effect of AMPK on NKA activity, NKA α subunit subcellular expression, downstream Src-NLRP3-IL-1β signaling, autophagy, and mitochondrial function and examined possible pathways involved. We also evaluated and compared the AMPK-activating effects of metformin with those of the UA-lowering agent febuxostat in an HUA rat model.

## Materials and methods

### Materials and reagents

Human primary renal PTECs and cell culture medium were purchased from ScienCell (San Diego, CA, USA). UA and oxonic acid (OA) were purchased from Sigma (St. Louis, MO, USA). An IL-1β ELISA kit was purchased from eBioscience (San Diego, CA, USA). A Cell Surface Protein Isolation Kit was purchased from Pierce (Rockford, IL, USA). Anti-NKA α1, Src/phosphorylated (p)-Src, AMPK/pAMPK, CIAS1/NALP3, peroxisome proliferator-activated receptor γ-coactivator-1α (PGC-1α), beclin-1, SQSTM1/p62, lysosome-associated membrane protein 2 (LAMP2), and uncoupling protein 2 (UCP2) antibodies and a reactive oxygen species (ROS) production assay kit were obtained from Abcam (Cambridge, UK). Anti-mammalian target of rapamycin (mTOR)/pmTOR and forkhead box O3a (FoxO3a)/pFoxO3a antibodies were obtained from Cell Signaling Technology (Danvers, MA, USA). An anti sirtuin type 1 (Sirt1) antibody was obtained from Millipore (Burlington, MA, USA). An anti-IL-1β antibody was obtained from Santa Cruz Biotechnology (Dallas, TX, USA). An anti-LC3 antibody was obtained from Novus (Littleton, CO, USA). Secondary antibodies were purchased from Sinobio (Shanghai, China). An NKA activity kit, a mitochondrial isolation kit, a mitochondrial membrane potential (MMP) assay kit, an aldolase assay kit and respiratory complex I, II, III, IV, and V activity kits were purchased from Genmed (Shanghai, China). LysoTracker Red was purchased from Beyotime (Shanghai, China). UA, AMP, and ATP measurement kits were purchased from Jiwei Biological Technology (Shanghai, China). An annexin V/propidium iodide (PI) flow cytometry assay kit was obtained from Becton Dickinson (San Jose, CA, USA). mtDNA and 18SRNA primers were obtained from Invitrogen (Carlsbad, CA, USA). A Cell Counting Kit-8 (CCK-8) kit was purchased from Dojindo Laboratories (Kumamoto-ken, Kyushu Island, Japan). Febuxostat was kindly provided by Jiangsu Hengrui Medicine Co., Ltd. (Shanghai, China). Metformin was purchased from MedChemExpress (Monmouth Junction, NJ, USA).

### Animal model and measurement

Twenty 8-week-old male-specific pathogen-free SD rats weighing 200 to 250 g were bred at the animal center of Shanghai Rat & Mouse Biotech Co., Ltd. The rats were divided into four groups (*n* = 5 in each group): (1) the Cont group, (2) the OA group (750 mg/kg/day OA for 8 weeks), and (3) the OA + Feb group (750 mg/kg/day OA for 8 weeks + 3 mg/kg/day Feb for 4 weeks starting at the 5th week), and 4) the OA + Met group (750 mg/kg/day OA for 8 weeks + 250 mg/kg/day Met for 4 weeks starting at the 5th week). The rats were sacrificed at the end of the 8th week. Twenty-four hour urine samples were collected from the rats with metabolic cages. Serum UA (SUA), urinary UA (UUA), blood urea nitrogen (BUN), serum creatinine (Scr), urinary creatinine (Ucr), serum sodium (SNa), urinary sodium (UNa), serum potassium (SK) and urinary potassium (UK) concentrations were determined by an enzymatic colorimetric assay method using a fully automatic chemistry analyzer (MODULAR D/P, Roche). Urinary albumin (UALB) was measured using the sulfosalicylic acid method. The urinary albumin to creatinine ratio (UACR) was calculated as UALB/Ucr. Fractional excretion of uric acid (FEUA) was calculated as (UUA × Scr)/(SUA × Ucr) × 100 and expressed as a percentage. Fractional excretion of sodium (FENa) was calculated as (UNa × Scr)/(SNa × Ucr) × 100 and expressed as a percentage. Fractional excretion of potassium (FEK) was calculated as (UK × Scr)/(SK × Ucr) × 100 and expressed as a percentage.

Fresh renal cortex and liver tissues were collected at the time of sacrifice and then subjected to AMP, ATP, and UA measurements according to the manufacturer’s instructions. Some tissues were stained with hematoxylin-eosin (HE) or Masson’s trichrome stain for light microscopy. The rest of the tissues were stored at −80 °C for immunoblotting assays. All animal procedures were performed in accordance with the National Institutes of Health guidelines (NIH Pub. No. 85–23, revised 1996) and were approved by the Animal Care and Use Committee of Shanghai Rat & Mouse Biotech Co., Ltd.

### UA stimulation and RNA sequencing in PTECs

UA-containing (100 μg/mL) cell culture medium was added to stimulate 2 × 10^5^ preseeded PTECs. After 0, 24, and 48 h of incubation, the supernatants were discarded, and 200 μL of TRIzol reagent (Invitrogen, USA) was added to each of the triplicates for the three groups.

Total RNA was extracted by using TRIzol and a miRNeasy Mini Kit (Qiagen) according to the manufacturer’s protocol with DNase treatment. RNA quality was evaluated with an Agilent 2100QC Bioanalyzer (Agilent Technologies). All RNA samples had RNA integrity numbers (RINs) greater than 8.0. Briefly, 2 μg of total RNA was subjected to ribosomal RNA removal using a Ribo-Zero Magnetic kit (Epicenter, Illumina, USA), 300 ng of rRNA-depleted RNA was fragmented, and cDNA was synthesized using random primers (Takara, Japan) and SuperScript II polymerase (Invitrogen). Illumina TruSeq adaptors were added to the second strand and further amplified with P5/P7 primers. Fragments 350–500 bp in length were recovered with a gel extraction kit. All libraries were quantified and pooled at concentrations of 2 nM for sequencing on a NovaSeq system (150PE; Illumina, USA).

### RNA-seq data analysis

The raw read data from nine PTEC samples were filtered with Trimmomatic v0.22, mapped to the UCSC Hg19 human genome and annotated with the RefSeq database using TopHat v2.0.0. The mapping results were then processed using Cufflinks v2.0.4 for transcript assembly. The FPKM (Fragments per Kilobase per million mapped reads) value for each RefSeq-annotated gene was calculated and then transformed to a log2 scale. To avoid infinite values, a value of 1 was added to the FPKM value for each gene before log2 transformation.

Hierarchical clustering analysis (HCA) was performed using the Ward linkage method based on a distance matrix of the Pearson correlation of the samples in R (http://www.r-project.org/). Other analyses, such as expression heatmap analysis, Pearson correlation analysis, Student’s *t*-test, principal component analysis (PCA), and linear discriminant analysis (LDA), were performed using R (v3.3.3).

Functional enrichment analysis of the treatment-induced genes was completed with the Gene Ontology (GO) and Reactome pathway databases using Gene Set Enrichment Analysis (GSEA v3.0 beta).

### Detection of cell injury and mitochondrial function

Human primary PTEC culture and soluble UA preparation were performed as previously described^13^. Cell viability, apoptosis, MMP, and respiratory complex activity were analyzed according to the kit manufacturers’ instructions. Primers and probe sets for human mtDNA and 18SRNA were designed from known sequences in GenBank (Table 1) and were used in real-time PCR.

**Table 1:** Primers for real-time PCR

Gene	Accession number	Forward primer	Reverse primer
mtDNA	NC_012920	CCTCACTCATTTACACCAACCAC	TATAATCACTGCCCGCTCA
18SRNA	NR_003286	GCGGTTCTATTTTGTTGGTTTT	ACCTCCGACTTTCGTTCTTG

### Western blotting

Western blot analysis was conducted as described previously^13^ with antibody dilutions of 1:2000 (beclin-1 and LC3), 1:1000 (p62, CIAS1/NALP3, pSrc, Src, pmTOR, mTOR, pFoxO3a, and FoxO3a), and 1:500 (AMPK, pAMPK, LAMP2, UCP2, PGC-1α, Sirt1, and IL-1β).

### NKA enzymatic activity measurements in PTECs and in rat renal cortex tissue

NKA activity in cultured PTECs was measured with an NKA activity kit^14^. NKA activity specifically refers to ouabain-sensitive NKA activity and was defined as the amount of NKA required to oxidize 1 mol of NADH into nicotinamide adenine dinucleotide (NAD) at 37 °C and pH 7.5 per milligram of protein per minute. All procedures were performed according to the manufacturer’s instructions.

Fifty micrograms of fresh rat renal cortex tissues were collected, 50 mL of 0.01 mol/L PBS (pH 7.4) was added, and the renal tissue was homogenized using a homogenizer. The samples were centrifuged at 8000 × *g* for 10 min at 4 °C. The supernatant was collected and subjected to NKA activity measurement. NKA activity was obtained by measuring inorganic phosphate (Pi) release using an NKA activity kit (ToYongBio, Shanghai) as per Forbush’s method^15^. The procedures were performed according to the manufacturer’s instructions. Readings were obtained at 660 nm. Enzyme-specific activity is expressed as 1 μmol of Pi released per milligram of protein per hour.

### Expression of the NKA α1 subunit in the cell membranes and lysosomes of PTECs and in the rat renal cortex

The abundance of the endogenous NKA α1 subunit on PTEC membranes was analyzed by determining surface biotinylation using a Cell Surface Protein Isolation Kit according to the manufacturer’s instructions. Surface proteins were eluted and processed for Western blotting as described previously^13^ using mouse anti-NKA α1 (1:500) as the primary antibody. The colocalization of NKA α1 and LysoTracker Red was evaluated by immunocytochemistry as previously described^16^ and according to the manufacturer’s instructions using an anti-NKA α1 antibody diluted 1:200 in staining buffer. Fluorescence was detected using an LSM 510 Meta confocal laser-scanning microscope (Leica, TCS-SP5, Solms, Germany). Western blotting of NKA α1 on the renal cortex was conducted with an anti-NKA α1 (1:1000) antibody.

### Detection of IL-1β, aldolase, AMP, and ATP in PTECs

The IL-1β and aldolase protein levels in culture supernatants were determined using commercial assay kits according to the manufacturers’ instructions. Cell lysates were collected, and intracellular ATP and AMP levels were measured using bioluminescence assay kits according to the manufacturer’s instructions.

### Statistical analysis

All data are expressed as the means ± standard deviations (SDs) unless otherwise specified. The statistical analysis was performed using SPSS v19.0 for Windows (SPSS, Inc., Chicago, IL, USA). Intergroup differences in continuous variables were assessed by multivariate analysis of variance (ANOVA). *P* < 0.05 was considered to indicate statistical significance.

## Results

### Enrichment analysis and liver kinase B1 (LKB1)-AMPK-mammalian target of rapamycin (mTOR) activation in UA-stimulated PTECs

**Fig. 1:**
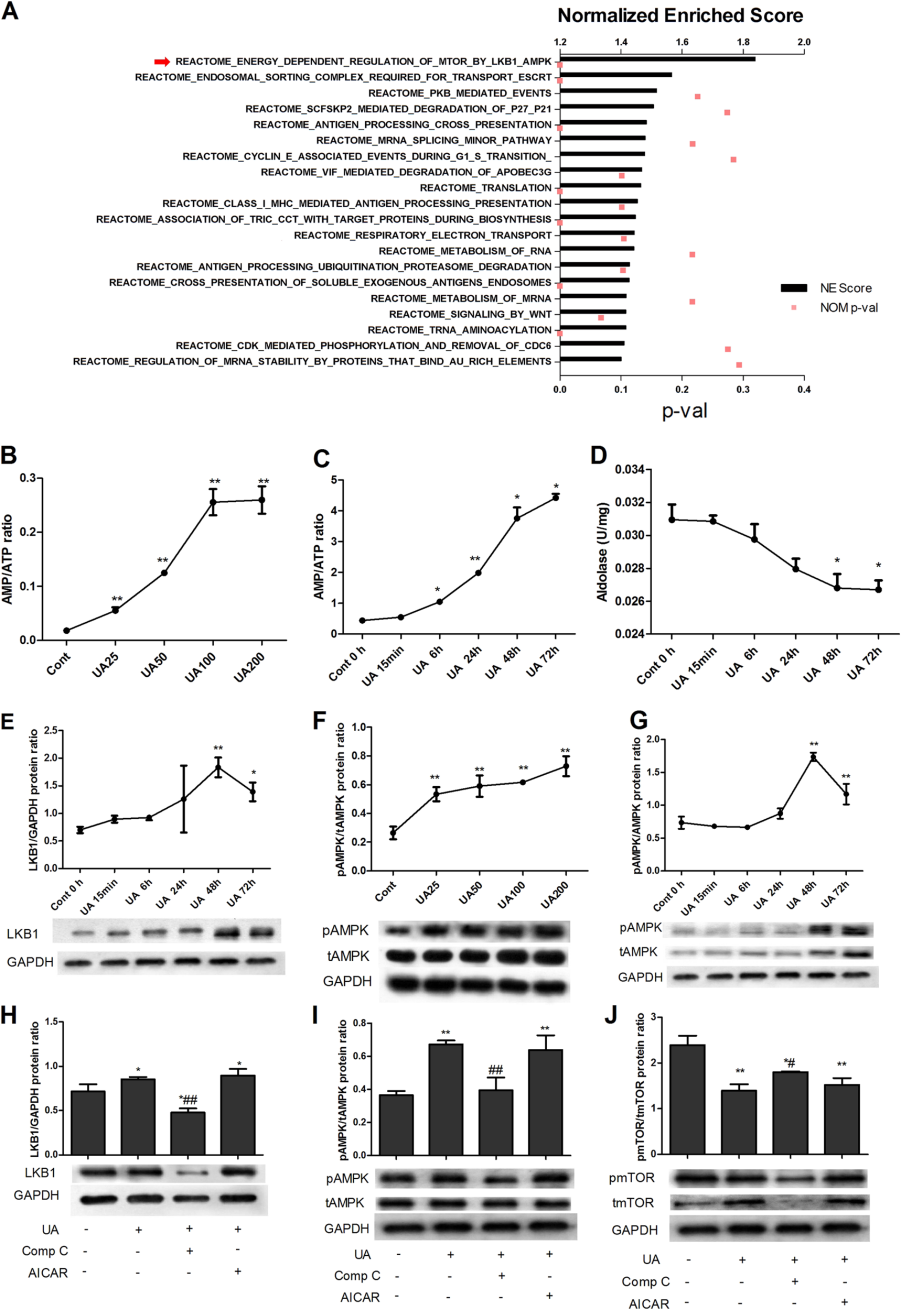
Enrichment analysis and analysis of LKB1-AMPK-mTOR activation in UA-stimulated PTECs. Transcriptomic analysis of UA-treated and untreated PTECs showed a total of 905 differentially expressed genes (DEGs) (FDR < 0.05) in UA-treated cells compared to control cells. Reactome-based Gene Set Enrichment Analysis (GSEA) was used to reveal the signaling pathways induced by UA treatment (**a**). Functional annotation of RNA-seq data revealed that energy-dependent regulation of the LKB1/AMPK/mTOR pathway was the most abundantly enriched pathway in UA-treated PTECs (**a**). UA stimulation (25 μg/mL, 50 μg/mL, 100 μg/mL, and 200 μg/mL) for 48 h increased intracellular AMP/ATP ratios (**b**) and AMPK activation (**f**) in PTECs in a dose-dependent manner. UA at 100 μg/mL (15 min, 6 h, 24 h, 48 h, and 72 h) increased the intracellular AMP/ATP ratio (**c**) and reduced aldolase levels (**d**) in a time-dependent manner. UA increased LKB1 (**e**) and AMPK activation (**g**) in a time-dependent manner, with maximal activation at 48 h, but activation started to decrease after 72 h. The AMPK inhibitor Comp C (20 μM) or the AMPK activator AICAR (0.1 mM) was added to cells for 1 h to inhibit or activate AMPK, respectively, before 48 h of stimulation with UA (100 μg/mL). UA significantly increased LKB1 (**h**) and AMPK activation (**i**) and reduced mTOR phosphorylation (**j**) in PTECs. Comp C significantly reduced LKB1 and AMPK activation and increased mTOR phosphorylation, whereas AICAR maintained LKB1 and AMPK activation and mTOR inhibition (**h**–**j**). *P < 0.05 vs. Cont, **P < 0.01 vs. Cont, ^#^*P* < 0.05 vs. UA, ^##^*P* < 0.01 vs. UA

To identify the UA-targeted pathway, we performed transcriptomic analysis on UA-treated and untreated PTECs. A total of 905 differentially expressed genes (DEGs) (FDR < 0.05) were observed in the UA-treated cells compared to the control cells after 24 h. Reactome-based Gene Set Enrichment Analysis (GSEA) was used to reveal the signaling pathways induced by UA treatment (Fig. 1a). Functional annotation of the RNA-seq results revealed that energy-dependent regulation of the LKB1/AMPK/mTOR pathway was the most abundantly enriched pathway in UA-treated proximal tubular epithelial cells (PTECs).

As LKB1/AMPK signaling has been shown to be activated depending on the AMP/ATP ratio or by aldolase^17^, we further examined the dose- and time-dependent effects of UA on energy metabolism molecules, including AMP/ATP (specifically, the ratio between the two molecules), aldolase, LKB1, and AMPK. UA stimulation (25 μg/mL, 50 μg/mL, 100 μg/mL, and 200 μg/mL) for 48 h increased the intracellular AMP/ATP ratios (Fig. 1b) and the activation of AMPK (phosphorylated AMPK/total AMPK, Fig. 1f) in PTECs in a dose-dependent manner. UA at 100 μg/mL (15 min, 6 h, 24 h, 48 h and 72 h) increased the intracellular AMP/ATP ratio (Fig. 1c) and reduced aldolase levels (Fig. 1d) in a time-dependent manner. UA increased LKB1 (Fig. 1e) and AMPK activation (Fig. 1g) in a time-dependent manner, with maximal activation at 48 h, but the activation of these molecules started to decrease after 72 h. We selected 100 μg/mL UA and 48 h of stimulation for the subsequent experiments.

To elucidate whether the activation of AMPK by UA is a compensatory protective response or is a direct reflection of injury, we added the AMPK inhibitor Compound C (Comp C, 20 μM) or the AMPK activator AICAR (0.1 mM) 1 h before the 48 h stimulation with UA (100 μg/mL) to inhibit or activate AMPK, respectively. UA significantly increased LKB1 (Fig. 1h) and AMPK activation (Fig. 1i) and reduced mTOR phosphorylation (Fig. 1j) in PTECs. Comp C significantly reduced LKB1 and AMPK activation and increased mTOR phosphorylation, whereas AICAR maintained LKB1 and AMPK activation and mTOR inhibition (Fig. 1h–j).

### AMPK activation increased NKA activity, alleviated lysosomal NKA degradation, and increased NKA signaling in UA-stimulated PTECs

**Fig. 2:**
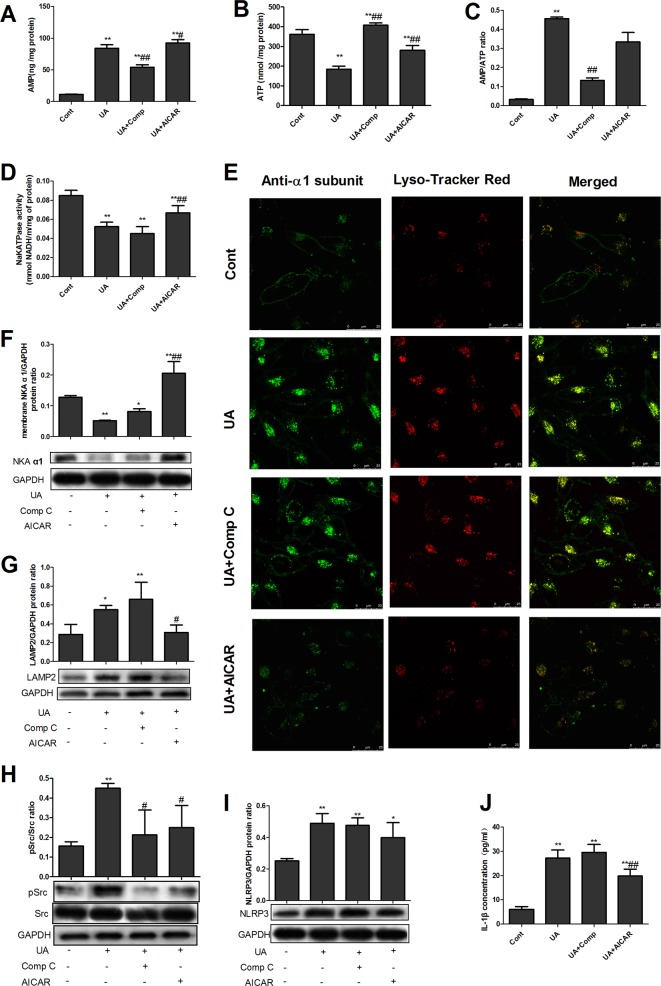
AMPK activation increased NKA activity and reduced NKA signaling in UA-stimulated PTECs. UA significantly reduced intracellular ATP levels (**b**) and increased AMP levels (**a**) and the AMP/ATP ratio (**c**) in PTECs. Comp C significantly increased intracellular ATP levels and reduced AMP levels and the AMP/ATP ratio, whereas AICAR increased ATP and AMP levels but had no effect on the AMP/ATP ratio. UA reduced NKA activity (**d**), increased NKA lysosomal degradation (**e**), and thus reduced NKA membrane surface expression (**f**). AICAR treatment with UA stimulation significantly reduced NKA expression in lysosomes and enhanced NKA activity and the cell surface expression of the NKA α1 subunit (**d**–**f**) compared with UA stimulation alone. UA also significantly increased the expression of the lysosomal marker LAMP2, while AICAR treatment with UA reduced LAMP2 expression compared with UA alone (**g**). UA significantly induced Src activation (**h**) and increased NLRP3 (**i**) and IL-1β (**j**) expression. Comp C significantly reduced UA-induced Src activation (**h**) but not NLRP3 (**i**) and IL-1β (**j**) activation, whereas AICAR significantly reduced Src (**h**) and IL-1β (**j**) activation. AICAR also showed a tendency to reduce UA-induced NLRP3 overexpression (**i**). *P < 0.05 vs. Cont, **P < 0.01 vs. Cont, ^#^*P* < 0.05 vs. UA, ^##^*P* < 0.01 vs. UA

We then used NKA activity and intracellular staining assays to identify whether AMPK activation plays a protective or detrimental role in the UA-induced impairment of NKA expression and signaling. The results showed that UA significantly reduced intracellular ATP levels (Fig. 2b) and increased AMP levels (Fig. 2a) and the AMP/ATP ratio (Fig. 2c) in PTECs. Comp C significantly increased intracellular ATP levels and reduced AMP levels and the AMP/ATP ratio, whereas AICAR increased ATP and AMP levels but had no effect on the AMP/ATP ratio. UA reduced NKA activity (Fig. 2d), increased NKA lysosomal degradation (Fig. 2e), and thus reduced NKA surface expression (Fig. 2f). AICAR treatment with UA stimulation significantly reduced NKA expression in lysosomes, enhanced NKA activity and enhanced the cell surface expression of the NKA α1 subunit (Fig. 2d–f) compared with UA stimulation alone. UA also significantly increased the expression of the lysosomal marker LAMP2, while AICAR treatment with UA reduced LAMP2 expression compared with UA alone (Fig. 2g).

UA significantly induced Src activation (p-Src/Src, Fig. 2h) and increased NLRP3 (Fig. 2i) and IL-1β (Fig. 2j) expression. Comp C significantly reduced UA-induced Src activation but not NLRP3 and IL-1β activation; in contrast, AICAR significantly reduced Src and IL-1β activation. AICAR also showed a tendency to reduce UA-induced NLRP3 overexpression (Fig. 2h–j).

### AMPK alleviated UA-induced apoptosis and autophagy in UA-stimulated PTECs

**Fig. 3:**
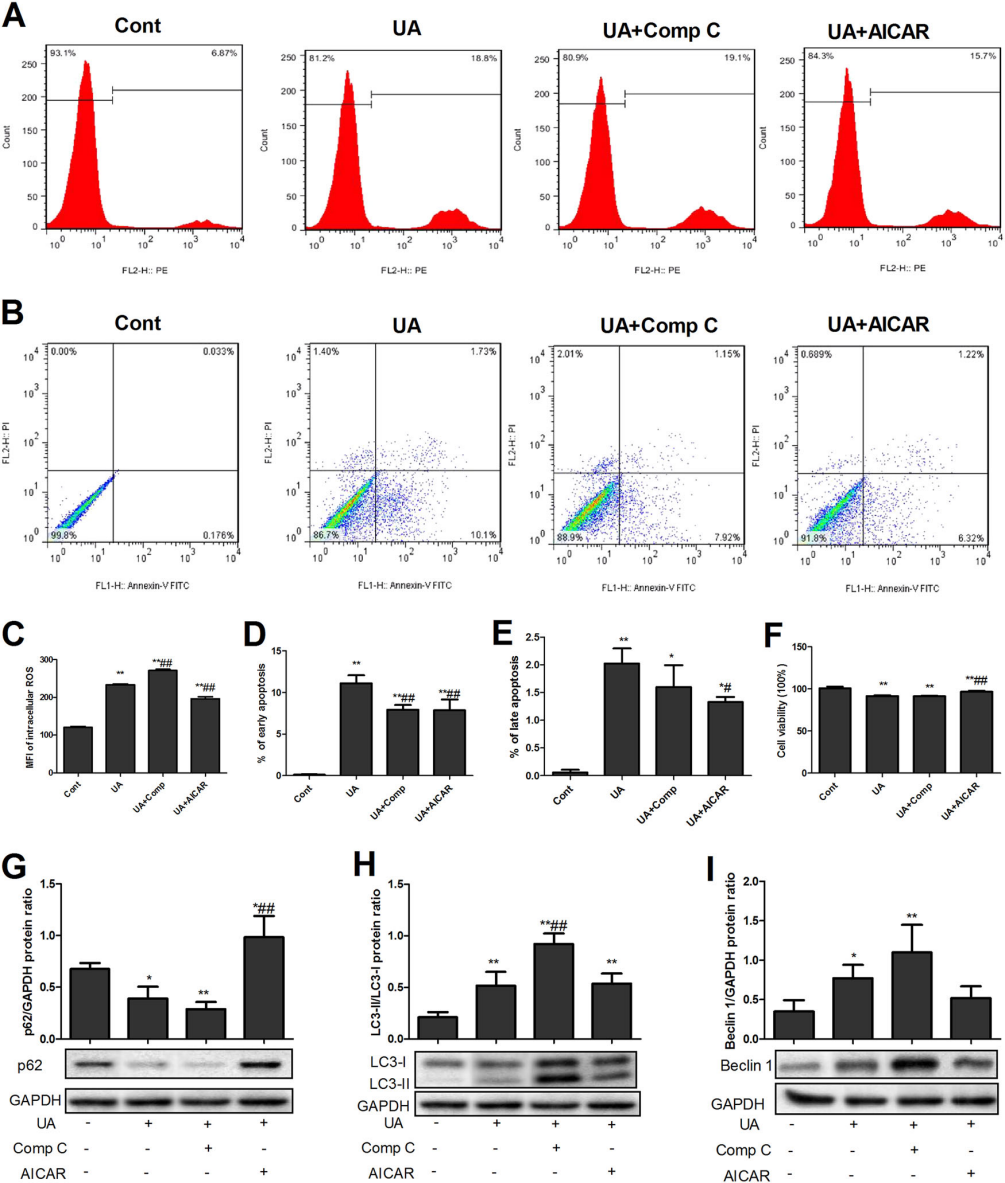
AMPK alleviated UA-induced apoptosis and autophagy in UA-stimulated PTECs. UA significantly increased reactive oxygen species (ROS) production (**a**, **c**) and early and late apoptosis (**b**, **d**, **e**); reduced cell viability (**f**); induced autophagy, as indicated by reduced p62 levels (**g**); and increased the LC3-II/LC3-I ratio (**h**) and beclin-1 levels (**i**). AICAR significantly attenuated ROS production (**a**, **c**), reduced early and late apoptosis (**b**, **d**, **e**), increased cell viability (**f**) and p62 levels (**g**), and showed a tendency to reduce beclin-1 levels (**i**) and the LC3-II/LC3-I ratio (**h**). Comp C increased ROS (**a**, **c**) and the LC3-II/LC3-I ratio (**h**). *P < 0.05 vs. Cont, **P < 0.01 vs. Cont, ^#^*P* < 0.05 vs. UA, ^##^*P* < 0.01 vs. UA

We have previously demonstrated that UA induces NKA-mediated ROS production, apoptosis and autophagy^6^. Here, we showed that AMPK activation by AICAR significantly alleviated UA-induced ROS production (Fig. 3a, c), reduced early and late apoptosis (Fig. 3b, d, e), and increased cell viability (Fig. 3f) and p62 (Fig. 3g) and showed a tendency to reduce beclin-1 levels (Fig. 3h) and the LC3-II/LC3-I ratio (Fig. 3i). Comp C increased ROS (Fig. 3a, c) and the LC3-II/LC3-I ratio (Fig. 3h).

### AMPK alleviated UA-induced mitochondrial dysfunction and phosphorylation of forkhead box O3a (FoxO3a) in PTECs

**Fig. 4:**
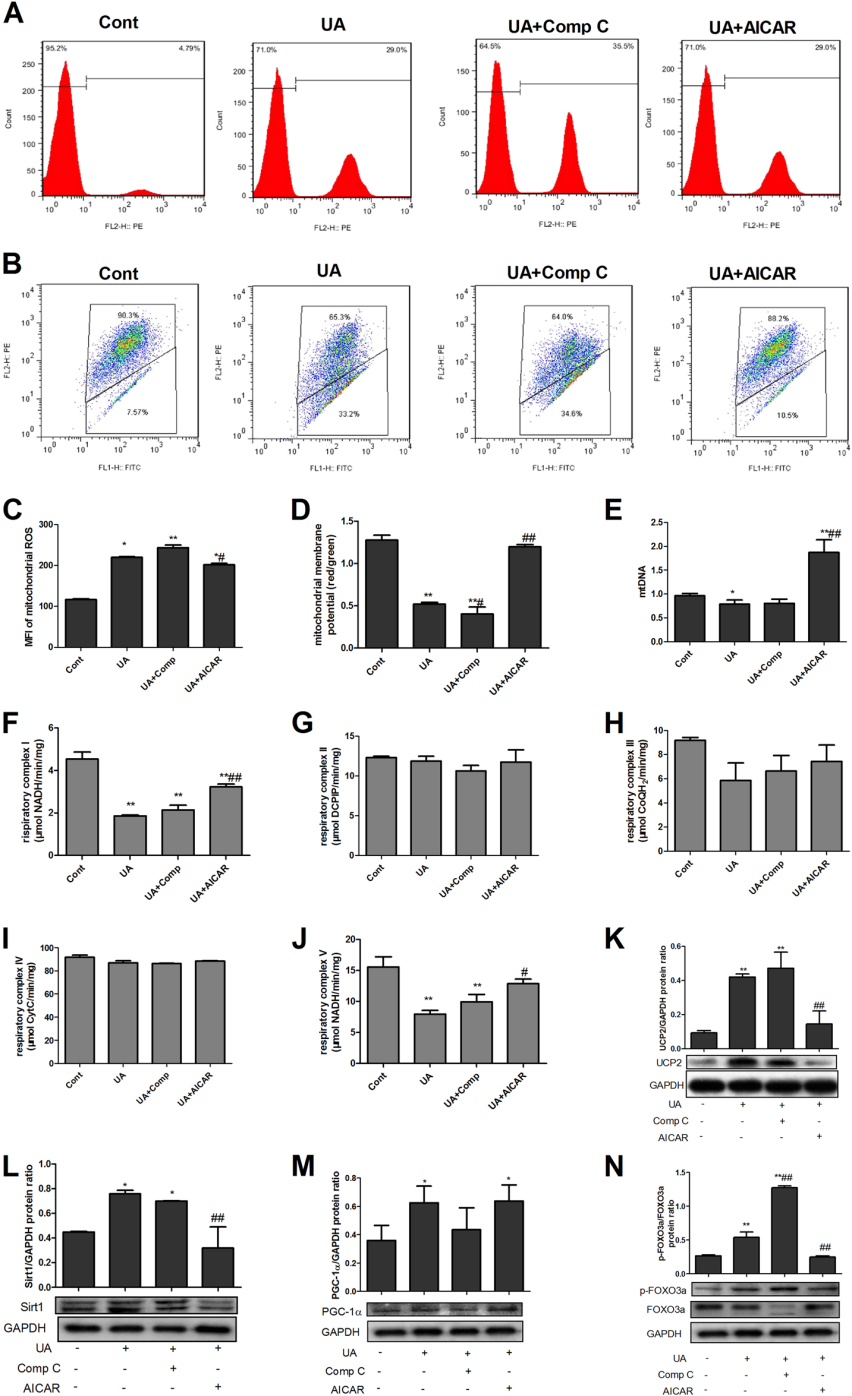
AMPK alleviated UA-induced mitochondrial dysfunction and Sirt1/FoxO3a changes in PTECs. UA significantly increased mitochondrial ROS production (**a**, **c**); reduced mitochondrial membrane potential (MMP) (**b**, **d**), mitochondrial DNA (mtDNA) copy numbers (**e**), and mitochondrial respiratory complex I (**f**) and V (**j**) activity; and increased UCP2 (K), Sirt1 (**l**), and PGC-1α expression (**m**) and FoxO3a phosphorylation (**n**). UA exerted no effect on mitochondrial respiratory complex II (**g**), III (**h**) and IV (**i**) activities. AICAR alleviated mitochondrial dysfunction by increasing MMP (**b**, **d**), mtDNA (**e**), and complex I (**f**) and V (**j**) activity and reducing mitochondrial ROS (**a**, **c**) and UCP2 expression (**k**). AICAR significantly reduced Sirt1 expression (**l**) and FoxO3a phosphorylation (**n**). Comp C significantly increased FoxO3a phosphorylation (**n**). *P < 0.05 vs. Cont, **P < 0.01 vs. Cont, ^#^*P* < 0.05 vs. UA, ^##^*P* < 0.01 vs. UA

We previously demonstrated that UA induces NKA-mediated mitochondrial dysfunction^6^. In the current study, we further found that UA induced Sirt1 (Fig. 4l) and PGC-1α (Fig. 4m) overexpression and FoxO3a phosphorylation (Fig. 4n). UA exerted no effect on mitochondrial respiratory complex II (Fig. 4g), III (Fig. 4h) and IV (Fig. 4i) activities. AMPK activation by AICAR alleviated UA-induced mitochondrial dysfunction by increasing MMP (Fig. 4b, d), mtDNA (Fig. 4e), and complex I (Fig. 4f) and V (Fig. 4j) activity and by reducing mitochondrial ROS (Fig. 4a, c) and UCP2 expression (Fig. 4k). AICAR also significantly reduced Sirt1 expression (Fig. 4l) and FoxO3a phosphorylation (Fig. 4n). Comp C significantly increased FoxO3a phosphorylation (Fig. 4n).

### AMPK alleviated HUA-induced NKA signaling impairment and renal tubular injury in vivo

**Fig. 5:**
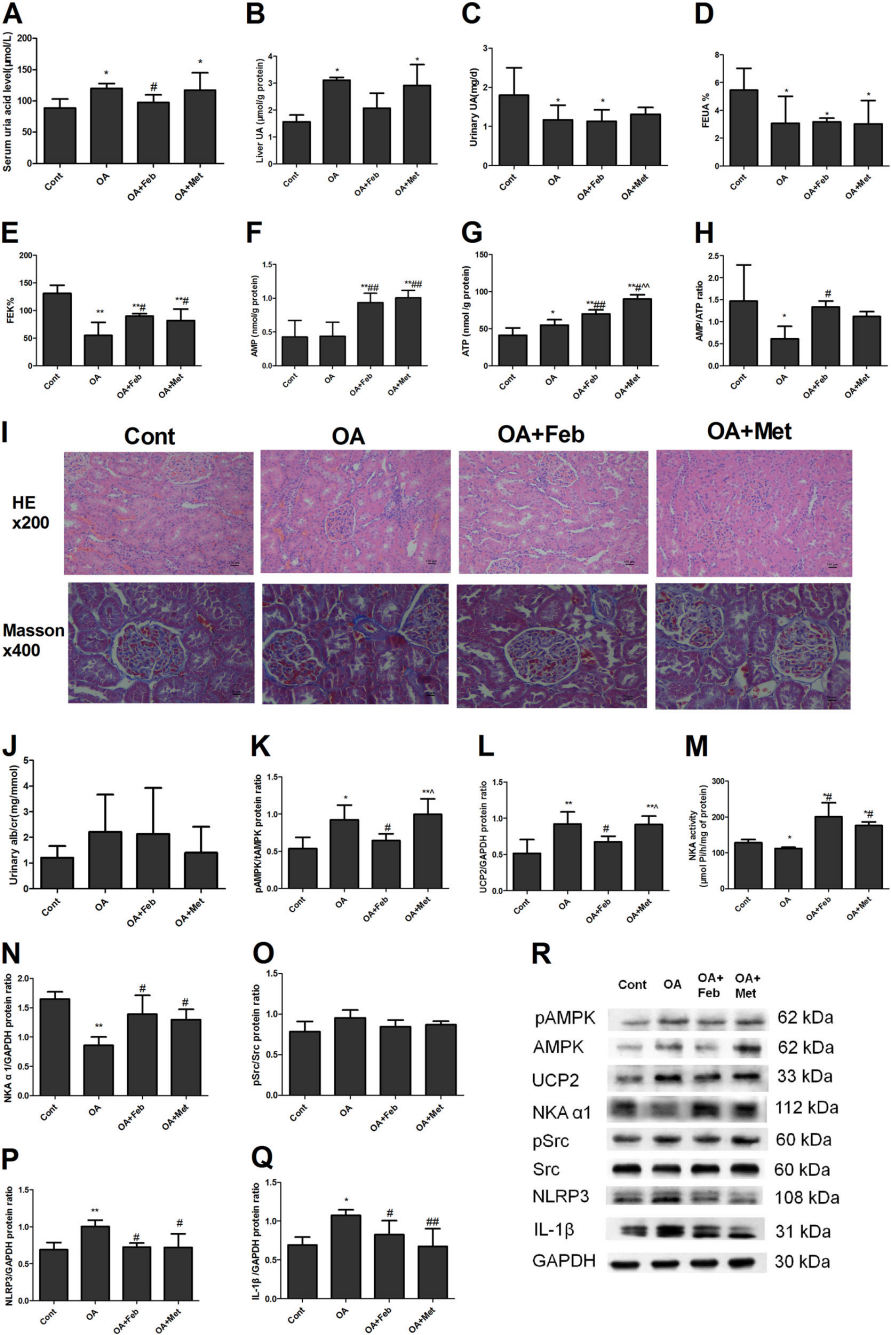
AMPK alleviated HUA-induced NKA impairment and renal tubular injury in vivo. Male SD rats were administered gastric oxonic acid (OA, 750 mg/kg/day) for 8 weeks and administered Feb (3 mg/kg/day) or Met (250 mg/kg/day) for 4 weeks starting at the 5th week. OA rats showed significantly increased serum (**a**) and liver (**b**) UA levels; reduced 24 h UUA levels (**c**), FEUA (**d**), FEK (**e**); and reduced renal cortex AMP/ATP ratios (**h**). OA rats displayed renal tubular dilation with epithelial atrophy and interstitial infiltration of inflamed cells (**i**). OA rats also developed increased AMPK activation (**k**, **r**) and UCP2 levels (**l**, **r**), reduced NKA activity (**m**) and reduced NKA α1 subunit expression (**n**, **r**). OA also enhanced NLRP3 (**p**, **r**) and IL-1β (**q**, **r**) expression in the renal cortex. OA rats showed a tendency of increased UACR (**j**) and Src activation (p-Src/Src, **o**, **r**), but with no statistical significances. Metformin did not reduce serum UA levels (**a**) in OA-administered rats but demonstrated effects similar to those of febuxostat in that it increased FEK (**e**), AMP levels (**f**), NKA activity (**m**), and NKAα1 subunit expression (**n**, **r**) and attenuated NLRP3 (**p**, **r**) and IL-1β (**q**, **r**) expression. Metformin caused greater AMPK activation (**k**, **r**) and ATP elevation (**g**) and less UCP2 alleviation (**l**, **r**) than febuxostat in OA-administered rats. *P < 0.05 vs. Cont, **P < 0.01 vs. Cont, ^#^*P* < 0.05 vs. OA, ^##^*P* < 0.01 vs. OA. ^*P* < 0.05 vs. OA + Feb, ^^*P* < 0.01 vs. OA + Feb

We further validated and compared the protective effect of metformin-mediated AMPK activation with that of the UA-lowering agent febuxostat in HUA rats. Male SD rats were administered gastric oxonic acid (OA, 750 mg/kg/day) for 8 weeks and administered Feb (3 mg/kg/day) or Met (250 mg/kg/day) for 4 weeks starting at the 5th week. OA rats showed significantly increased serum and liver UA levels, reduced FEUA, FEK and 24 h UUA levels, and reduced renal cortex AMP/ATP ratios (Fig. 5a–e, h). OA rats displayed renal tubular dilation with epithelial atrophy and interstitial infiltration of inflamed cells (Fig. 5i). OA rats also developed increased AMPK activation and UCP2 levels, reduced NKA activity and NKA α1 subunit expression, and enhanced NLRP3 and IL-1β expression in the renal cortex (Fig. 5k–n, p–r). OA rats showed a tendency of increased UACR (Fig. 5j) and Src activation (p-Src/Src, Fig. 5o, r), but with no statistical significances. The rat BUN, Scr, FENa and renal UA levels of all the groups did not differ (data not shown). Metformin did not reduce serum UA levels in OA-administered rats but demonstrated effects similar to those of febuxostat in that it increased FEK, AMP levels, NKA activity, and NKAα1 subunit expression, and reduced NLRP3 and IL-1β expression (Fig. 5e–f, m–n, p–r). Metformin caused more AMPK activation (Fig. 5k, r) and ATP elevation (Fig. 5g) but less UCP2 reduction (Fig. 5l, r) than febuxostat in OA-treated rats.

## Discussion

In our previous study, we demonstrated the contribution of impaired NKA to HUA-induced renal tubular inflammation^6^. In the current study, we further verified by GSEA that the LKB1/AMPK/mTOR pathway was the most abundantly enriched pathway in UA-stimulated PTECs. We also demonstrated that UA significantly activated AMPK in the renal cortex of HUA rats and in UA-stimulated PTECs. The impairment of NKA signaling and the occurrence of NKA-mediated mechanisms of cell injury, such as ROS production, autophagy, apoptosis, mitochondrial dysfunction, and the inflammatory response, could be significantly alleviated by sustained AMPK activation.

We first screened possible targeted pathways involved in HUA-induced renal tubular injury using RNA-seq in a cell model, and the GSEA results revealed that the LKB1/AMPK/mTOR pathway was the most abundantly enriched pathway. We further validated and demonstrated the activation of AMPK in a high UA-stimulated in vitro PTEC model. LKB1 and AMPK activation increased gradually at first, peaked at 48 h, and started to decrease at 72 h under UA stimulation, suggesting a possible temporary compensatory response of LKB1/AMPK to counteract the detrimental effect of UA; however, at 72 h, AMPK activity started to decrease. LKB1 is activated by high AMP/ATP ratios, which are classic activators, and by the recently found aldolase^17^. We measured both AMP/ATP ratios and aldolase levels and demonstrated that the AMP/ATP ratio time-dependently increased and remained elevated at 72 h, while aldolase was reduced time-dependently until 48 h but did not continue to decrease after 48 h. Our findings suggest that UA may trigger the activation of LKB1/AMPK by increasing the AMT/ATP ratio and/or reducing aldolase levels, a possibility that needs further exploration. Similar to our findings, other researchers have also observed the activation of AMPK after 30 min of stimulation with 10 mg/dL and 15 mg/dL UA in rat pancreatic β-cells^18^ and after 24 h of stimulation with 15 mg/dL UA in skeletal muscle cells^19^ and in human monocytic THP-1 cells^20^. However, 16 h of stimulation with 0.2 mg/mL monosodium urate (MSU) crystals has been found to inhibit AMPK activation in macrophages^21^ and in a gouty arthritis animal model^21^. We have also examined whether soluble urate, but not the crystal form of MSU, exerted NLRP3-IL-1β-associated inflammatory effects in renal tubular^13^ and mesangial cells^22^. In the present study, we similarly used soluble UA rather than the crystal form. It is possible that different urate forms (soluble or crystal) could affect AMPK activation differently; this possibility needs further investigation.

We have previously demonstrated that the impairment of NKA function is one of the pivotal steps in HUA-induced renal tubular injury^6^ and that AMPK activation is observed in HUA-induced renal tubular injury^23^. In our present study, we further noted that AMPK activation significantly increased NKA activity by restoring NKA cell surface expression following AICAR treatment, whereas the AMPK inhibitor Comp C exerted the opposite effects. In HUA rats, although metformin-mediated AMPK activation did not significantly reduce serum UA, as febuxostat-mediated activation did, metformin treatment significantly alleviated NKA signaling impairment and renal tubular injury, effects that were not inferior to those of febuxostat. This result suggested that the renal tubular protective effect of AMPK activation by metformin in vivo did not depend on reductions in serum UA. Although metformin failed to reduce high UCP2 levels in OA-treated rats, it significantly increased renal ATP production compared with febuxostat, suggesting that AMPK could provide more energy for renal tubules, and further exerted a protective effect through restoration of NKA expression on the membrane. Large amounts of ATP are needed to enable NKA function and to maintain normal NKA activity. Similar results have been observed in canine kidney cells, in which the AMPK inhibitor Comp C increased NKA cell internalization and degradation^24^, whereas the AMPK activator metformin protected basolateral NKA in tubular cells from ischemia-induced cytoplasmic transport and degradation^11^. In L6 muscle cells, AICAR also increases NKA activity and expression on the cell membrane via NKA dephosphorylation at Ser18^12^. However, in lung epithelial H441 cells, AICAR inhibits NKA activity^25,26^ and mediates NKA internalization and degradation^27–29^. It is possible that renal tubular cells and muscle cells are energy-demanding cells, whereas lung tubular cells consume less energy.

As NKA is degraded in lysosomes and as AMPK reduces NKA expression in lysosomes, we studied mechanisms in vitro, examining apoptosis and autophagy levels in cells under HUA conditions with or without AMPK modulators. We observed that AMPK activation by AICAR significantly relieved high UA-induced apoptosis, autophagy and lysosomal marker changes. Recent studies have demonstrated that AMPK senses not only cellular glucose but also cellular stress and that this sensing of AMPK could be independent of the AMP/ATP ratio^17^; such sensing occurs on lysosomes and depends on the activation of LKB1 but can be triggered by aldolase during glucose deficiency^17^. We also noted that AMPK activation by AICAR did not reduce the AMP/ATP ratio, while Comp C significantly reduced the AMP/ATP ratio but did not exert any protective effect. We examined aldolase and LKB1 levels under UA stimulation and found that aldolase and LKB1 were activated, suggesting the activation of AMPK by aldolase/LKB1 under UA stimulation; however, this possibility needs further investigation. It is generally recognized that AMPK can inhibit mTOR complex 1 (mTORC1) activity^30^, releasing the inhibitory effect of mTORC1 and allowing autophagy to proceed^31^. Our GSEA analysis demonstrated enrichment of the LKB1/AMPK/mTOR pathway, and we observed reduced mTOR phosphorylation and increased autophagy in UA-stimulated cells accompanied by activation of AMPK. Although AICAR did not further change the extent of mTOR phosphorylation under UA stimulation, it significantly alleviated autophagy, while the AMPK inhibitor Comp C significantly increased mTOR phosphorylation and enhanced autophagy in high UA-stimulated PTECs. These findings suggest that mTOR is actively involved in the effect of AMPK on high UA-induced autophagy. Emerging evidence has identified a common location, the lysosome, as the site of activation for both mTORC1 and AMPK during the shift between starvation and nutrient repletion^32–34^. AMPK activation by metformin has been found to occur through the lysosomal pathway^35^. We also observed that UA increased the levels of the lysosome marker LAMP2 but that LAMP2 was significantly reduced by AICAR, suggesting that lysosomes might be the key organelles involved in the effect of AMPK on UA-induced NKA-associated injury. AMPK may regulate the stress signal for NKA degradation under UA stimulation, and mTOR may be a key regulator involved.

In the classic Whittam model, NKA is coupled with mitochondrial function^36^, and we demonstrated that supplementation with NKA restored mitochondrial function in high UA-stimulated PTECs in our previous study^6^. In the current study, we demonstrated that AMPK activation by AICAR significantly relieved UA-induced mitochondrial dysfunction, as demonstrated by increased mtDNA, mitochondrial complex I and V (ATP Synthase) activity, and ATP production and reduced mitochondrial ROS, MMP and UCP2 expression. We also studied regulators of mitochondrial biogenesis and found that UA significantly increased Sirt1, PGC-1α, and FoxO3a phosphorylation. AMPK activation significantly alleviated the increases in Sirt1 and FoxO3a phosphorylation, while Comp C exerted no effect on Sirt1 and significantly enhanced HUA-induced FoxO3a phosphorylation, suggesting the involvement of Sirt1/FoxO3a in AMPK-mediated regulation of mitochondrial function under high UA stimulation in renal tubules. As FoxO3a is one of the key regulators of autophagy/apoptosis^37,38^, we suggest that AMPK may act through FoxO3a to enable crosstalk between mitochondria and lysosomes.

**Fig. 6:**
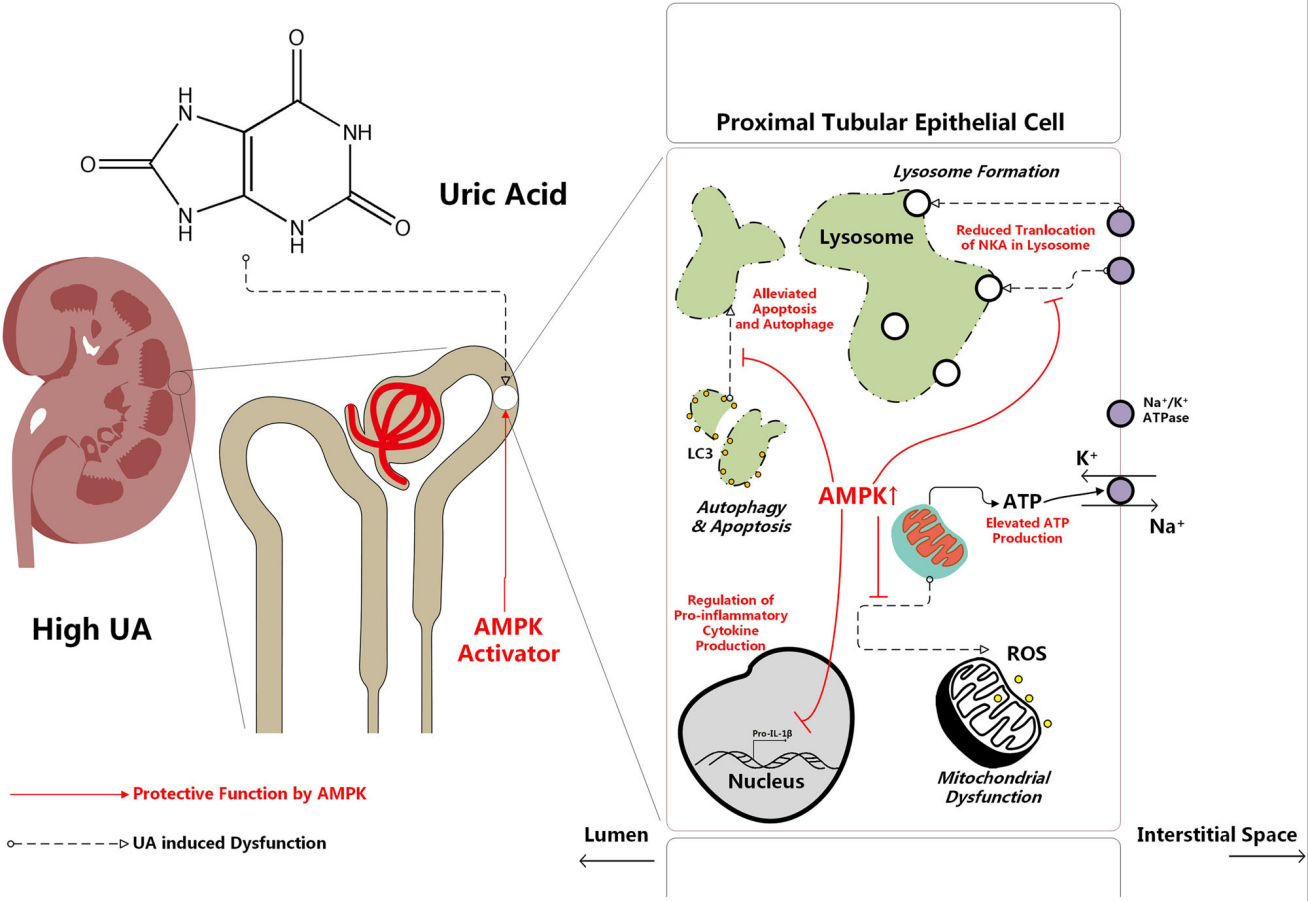
Scheme of the proposed mechanism of the protective effect of AMPK activation in HUA-induced renal injury. High UA induced translocation of membrane NKA to lysosomes, activated Src-NLRP3-IL-1β signaling downstream of NKA, increased autophagy and apoptosis, and increased mitochondrial dysfunction. However, UA also activated AMPK, which exerted a temporary compensatory effect for HUA-induced renal injury. Sustained AMPK activation reduced lysosomal NKA degradation and downstream inflammation and attenuated UA-induced autophagy, apoptosis, and mitochondrial dysfunction

In summary, we found that high UA-induced activation of AMPK temporarily compensated for HUA-induced renal injuries, including translocation of membrane NKA to lysosomes, NKA downstream Src-NLRP3-IL-1β signaling, increased autophagy and apoptosis, and mitochondrial dysfunction. Sustained AMPK activation reduced lysosomal NKA degradation and downstream inflammation and alleviated UA-induced autophagy, apoptosis and mitochondrial dysfunction (Fig. 6).
